# Supplementary material for: Counseling in Vape Shops: A Survey of Vape Shop Managers in Switzerland
Source: Int J Environ Res Public Health. 2021 Oct 15;18(20):10861. doi: 10.3390/ijerph182010861 (PMC8535370; doi:10.3390/ijerph182010861)
Supplement: Supplementary file 1 [file ijerph-18-10861-s001.zip › ijerph-1386957-supplementary.pdf]

Supplementary Material

Figure S1: perceived risk of vaporizer (ENDS) in a customer with stable coronary artery disease.

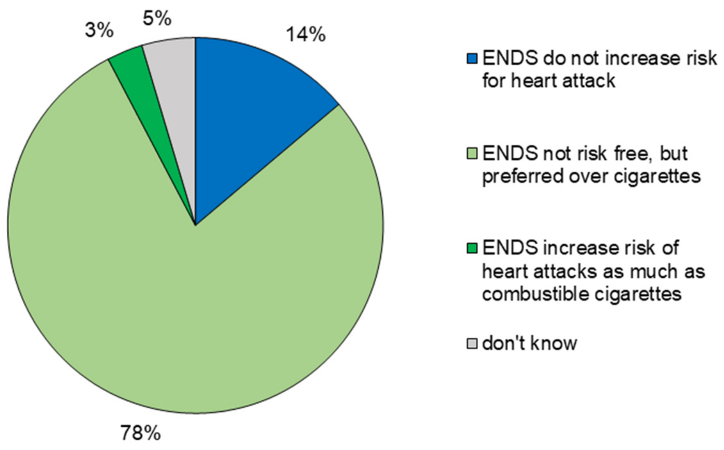

Figure S2: Box plot of perceived harmfulness of nicotine-containing products on a 0 – 9 scale. Abbreviations: HTP heated tobacco products; NRT nicotine replacement therapy; vaporizers/ENDS (electronic nicotine delivery system).

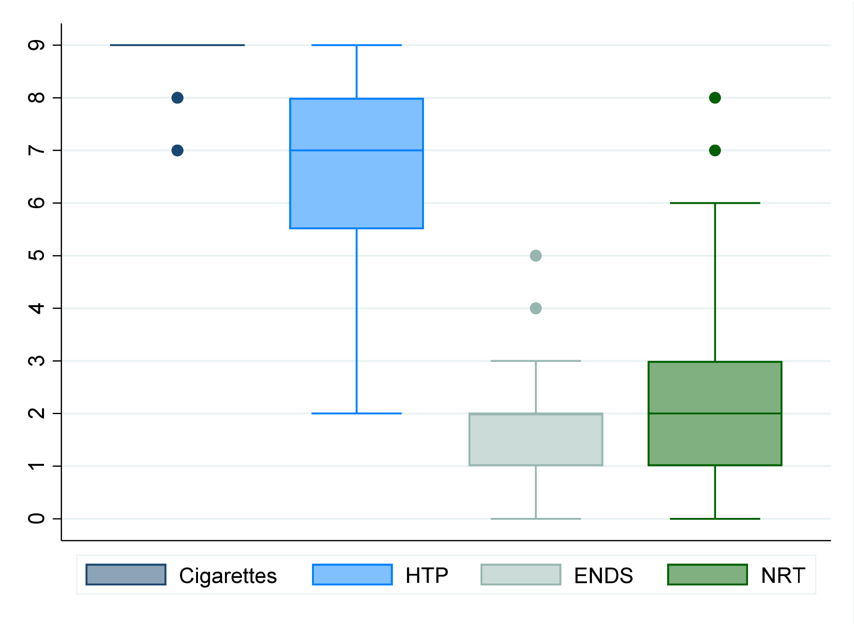

Table S1: Number of missing values for each variable with missing data.

Variable	*Missing values*
Sex	3
Age	7
Education	6
Experience working in vape shop	3
Smoking/Vaping Status	2
Role in shop	1
Selling Online	3
Selling Tobacco	1
Nicotine concentration (vignette 1)	3
Vignette 2 (Asthma)	2
Vignette 3.1 (What is your opinion about e-cigarettes that contain nicotine for persons after a heart attack?)	3
Vignette 3.2 (What do you suggest to him?)	5
Interest in training	2
Perceived harmfulness of nicotine containing products	3
